# Induced regulatory T cells for therapy: targeting *RBPJ* to enhance stability and function

**DOI:** 10.1038/s41392-025-02284-x

**Published:** 2025-06-23

**Authors:** Niklas Beumer, Michael Delacher

**Affiliations:** 1 Institute of Immunology, University Medical Center Mainz, Mainz, Germany; 2 Research Center for Immunotherapy, University Medical Center Mainz, Mainz, Germany

**Keywords:** Molecular engineering, Cell biology

In a recent study published in *Nature*, Chen et al.^1^ investigated the potential of *RBPJ* knock-out to improve stability and function of induced regulatory T cells (iTreg cells). This finding could further enhance the utilization of these cells for adoptive immunotherapy in immune-related diseases.

Regulatory T cells (Treg cells) play a crucial role in maintaining immune homeostasis. They are a subset of CD4-positive T cells that are selected as self-reactive clones during thymic maturation. The anti-inflammatory function of Treg cells is governed by the transcription factor forkhead-box P3 (FOXP3)^2^ and a thymus-induced epigenetic framework.^3^ As indispensable regulators of other immune cells, Treg cells ensure immune tolerance towards self and prevent autoimmune responses. Beyond their role in immune regulation, Treg cells also exhibit tissue-resident functions, migrating into organs to promote homeostasis and tissue repair upon damage.^4^

Treg cells possess anti-inflammatory and tissue homeostasis-promoting properties, making them an attractive option for adoptive cell therapy in patients with transplant rejection or graft-versus-host disease. In addition, the potential of Treg cells is investigated in various autoimmune diseases, including psoriasis, systemic lupus erythematosus, rheumatoid arthritis, and type 1 diabetes. To fully explore the therapeutic potential of Treg cells, it is essential to generate stable Treg cells in sufficient numbers. One promising approach for generating Treg cells is through ex vivo expansion of T cells in the presence of specific cytokines, such as tumor growth factor β (TGFβ) and interleukin 2 (IL-2). This strategy has been shown to induce the differentiation of T cells into functional Treg cells, offering a promising avenue for the development of Treg-cell-based therapies.

However, the clinical applicability of these in vitro induced Treg cells (iTreg cells) has been limited by their suboptimal long-term stability. To address this challenge, Chen et al.^1^ have identified a previously unknown mechanism that impairs iTreg cell stability and function. Their findings provide new insights into the complex processes governing iTreg cell function and stability, and offer an intriguing hypothesis for how iTreg cell generation may be improved in the future. This research has significant implications for the development of effective Treg-cell-based therapies for autoimmune diseases. The identification of the transcription factor RBPJ (Recombination Signal Binding Protein For Immunoglobulin Kappa J Region) as a negative regulator of iTreg cell stability highlights the importance of epigenetic regulation in the development and function of Treg cells.

Chen et al. utilized CRISPR screens and developed novel techniques termed intracellular RNA-seq (icRNA-seq), intracellular chromatin immunoprecipitation followed by sequencing (inChIP-seq), and intracellular assay for transposase-accessible chromatin using sequencing (inATAC-seq), which allow sequencing of cells stratified by intracellular protein expression. They found that FOXP3 levels, which they used as a proxy for iTreg stability, were associated with guide RNAs against *RBPJ*. This indicated that *RBPJ* negatively affects iTreg cell stability. *RBPJ* knock-out led to increased expression of Treg identity genes as well as immunosuppressive genes in iTreg cells. In line with this, Chen et al. observed that iTreg cells could suppress other T cells more potently after *RBPJ* knock-out. The effects of *RBPJ* deficiency on iTreg cell stability could even be shown under pro-inflammatory culture conditions involving multiple T cell receptor stimulations and exposure to tumor necrosis factor (TNF).

**Fig. 1:**
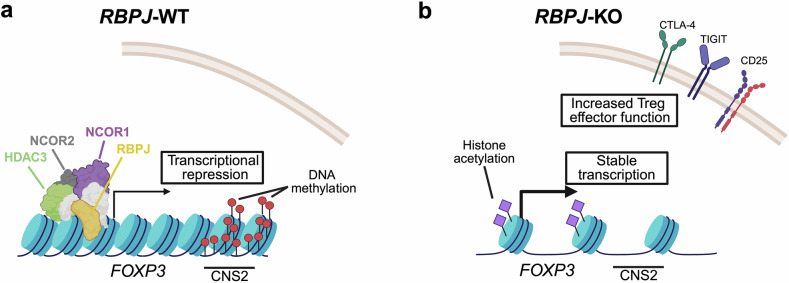
Model of how *RBPJ* knock-out can aid in the generation of stable iTreg cells. **a** Situation in wild-type iTreg cells. The RBPJ protein forms a repressive complex with other proteins, including NCOR1, NCOR2, and HDAC3. The enhancer region CNS2 in the first intron of the *FOXP3* gene is methylated, and *FOXP3* is not stably expressed or expression is lost during expansion. **b** Situation in iTreg cells upon *RBPJ* knock-out. Chromatin accessibility and histone acetylation around the *FOXP3* promoter are increased. CNS2 is demethylated. *FOXP3* is continuously transcribed, leading to a stable Treg cell identity. Treg cell effector genes are more strongly expressed, leading to enhanced Treg cell function. CNS conserved non-coding sequence, CTLA4 cytotoxic T-lymphocyte associated protein 4, FOXP3 forkhead-box P3, HDAC3 histone deacetylase 3, KO knock-out, NCOR nuclear co-receptor co-repressor, RBPJ recombination signal binding protein for immunoglobulin kappa J region, TIGIT T cell immunoreceptor with immunoglobulin and immunoreceptor tyrosine-based inhibitory motif domain, WT wild-type. The figure was generated using BioRender.com

The RBPJ protein participates in Notch signal transduction. If not activated, however, RBPJ forms a transcriptional repressor complex with other proteins like nuclear receptor co-repressor 1/2 (NCOR1/2) and the histone deacetylase 3 (HDAC3). Interestingly, Chen et al. found evidence that RBPJ-mediated effects on FOXP3 levels were independent from Notch signaling and were specific to the iTreg cell induction context. Mechanistically, Chen et al. established that RBPJ can directly bind to the *FOXP3* promoter. Furthermore, they provide evidence that the assembly of the repressor complex, including RBPJ, NCOR1/2, and HDAC3, is important for RBPJ-mediated inhibition of *FOXP3* expression. HDAC3 appears to be of particular importance in this context, as *HDAC3* knock-out reverted an observed impairment in iTreg cell differentiation upon *RBPJ* overexpression. In line with this, Chen et al. highlight several epigenetic traits that are linked to RBPJ-mediated suppression of *FOXP3*. For instance, they report that *RBPJ* knock-out leads to a reduction in DNA methylation at an important intronic *FOXP3* enhancer called the “conserved non-coding sequence 2” (CNS2). Furthermore, *RBPJ* knock-out increased histone 3 acetylation and chromatin accessibility around the *FOXP3* promoter, both of which represent epigenetic traits associated with increased transcription (Fig. 1).

Finally, Chen et al. explored the importance of *RBPJ* in vivo by testing iTreg cells with or without *RBPJ* knock-out in a graft-versus-host disease model in humanized mice. *RBPJ* knock-out rendered iTreg cells more stable in this setting. Furthermore, *RPBJ* knock-out in iTreg cells substantially improved the survival of affected mice. Survival was even comparable to the levels observed with “natural” Treg cells (i.e., purified CD4^+^CD127^low^CD25^high^ T cells) that did not have to be generated from other T cell types.

Altogether, these findings establish that RBPJ impairs the stability and function of Treg cells that are induced ex vivo. Its perturbation may thus be a suitable strategy to improve therapies based on iTreg cells, but clinical applications will likely require more extensive preclinical experimentation. So far, in vivo data are only available for a graft-versus-host disease setting in which iTreg cells were administered at disease onset. Experiments exploring other immune-related diseases with iTreg cell administration at later time points (i.e., at full-blown disease) will raise confidence in the therapeutic potential of iTreg cells with *RBPJ* knock-out. In addition, data from human patients will need to show that such cells are effective in a real-world clinical setting. There is a caveat related to the fact that *Rbpj* knock-out in mice was reported to have the opposite effect to what Chen et al. observed in human cells.^5^ Although Chen et al. discuss a potential reason for this, the discrepancy may indicate a yet unknown difference between humans and mice with respect to *RBPJ* and iTreg cells. Preclinical studies utilizing non-humanized mice thus need to exercise substantial caution in order to avoid conclusions that are opposite to what will be observed in human patients.

In conclusion, the results presented by Chen et al. highlight a promising strategy for the improvement of iTreg cell therapies. Furthermore, their CRISPR screens yielded a rich data set of potential *FOXP3* regulators that may lead to even more discoveries of how iTreg cell stability and function can be improved. Thus, Chen et al. have made a valuable contribution to potential therapies of immune-related diseases using iTreg cell adoptive transfer strategies.
